# Coinfection with chikungunya and Zika results in mild disease and distinct inflammatory response

**DOI:** 10.1038/s44298-025-00098-w

**Published:** 2025-02-11

**Authors:** Juliana Cardoso Alves, Lucas Sousa Magalhães, Priscila Lima dos Santos, Regina Adalva de Lucena Couto Ócea, Alejandra Debbo, Jaira Vanessa de Carvalho, Mauro Martins Teixeira, Suresh Mahalingam, Amelia Ribeiro de Jesus, Angela Maria da Silva, Roque Pacheco de Almeida, Camilla Natália Oliveira Santos

**Affiliations:** 1 Laboratório de Imunologia e Biologia Molecular, Universidade Federal de Sergipe, Aracaju, Brazil; 2 Programa de Pós-Graduação em Ciências da Saúde, Universidade Federal de Sergipe, Aracaju, Brazil; 3 Setor de Parasitologia e Patologia, Instituto de Ciências Biológicas e da Saúde, Universidade Federal de Alagoas, Maceió, Brazil; 4 Departamento de Educação em Saúde, Universidade Federal de Sergipe, Lagarto, Brazil; 5 Departamento de Medicina, Hospital Univeristário/EBSERH, Universidade Federal de Sergipe, Aracaju, Brazil; 6 Departamento de Bioquímica e Imunologia, Universidade Federal de Minas Gerais, Belo Horizonte, Brazil; 7 Institute for Biomedicine and Glycomics, Griffith University, Gold Coast, Australia; 8 Global Virus Network (GVN) Centre of Excellence in Arboviruses, Griffith University, Gold Coast, QLD Australia; 9 School of Pharmacy and Medical Sciences, Griffith University, Gold Coast, Australia

**Keywords:** Immunology, Virology, Medical research

## Abstract

Chikungunya (CHIKV) and Zika (ZIKV) viruses, both mosquito-borne, often circulate simultaneously, raising concerns about the effects of coinfection. This study evaluated cytokines, chemokines, and growth factors in 12 patients with concurrent CHIKV and ZIKV infections confirmed by RT-qPCR. Clinical data and 45 immune mediators were analyzed. Coinfected and monoinfected patients exhibited similar symptoms, although ZIKV-infected individuals experienced fewer instances of fever. No patients had persistent symptoms or required hospitalization. Chemokines CCL5, CXCL1, and CXCL10 were elevated across all groups. CHIKV-infected patients showed higher levels of CCL2, CCL4, EGF, CXCL12, and IFN-α compared to controls, while IL-1RA, IL-8, and IFN-γ were elevated in both CHIKV and coinfected groups. SCF was elevated only in the ZIKV group. Overall, CHIKV and ZIKV coinfection presented mild clinical symptoms similar to monoinfections and demonstrated a moderate inflammatory response.

## Introduction

Re-emerging arboviruses including chikungunya, Zika, Rift Valley Fever, West Nile viruses, pose significant global health concerns due to their potential for sudden outbreaks and rapid spread, driven by factors such as global travel, urbanization, and climate change^1^. Chikungunya and Zika viruses (CHIKV and ZIKV) are transmitted by the same mosquito vectors, primarily from the *Aedes* genus^2^. Initially reported in Africa, CHIKV has since spread to over 120 countries, and ZIKV to over 80, largely in tropical and subtropical regions^3^. This expansion has been facilitated by viral adaptability to mosquito species such as *Aedes albopictus*, which can thrive in a wide range of climates.

Both CHIKV and ZIKV can lead to mild manifestations, such as fever, exanthema, and myalgia. However, severe manifestations can occur, including chronic debilitating arthralgia in CHIKV-infected individuals, congenital abnormalities in ZIKV-infected newborns, and neurological disorders for both viruses^4–6^. The interplay between the host immune response, viral infection, and tropism is a crucial factor in the differences observed among clinical manifestations. CHIKV and ZIKV infections in humans trigger robust innate and adaptive immune responses, including the activation of type I interferons, T cells, and the production of neutralizing antibodies^7^. However, there are differences in cytokine profiles and cellular responses that reflect their distinct pathogenesis and tissue targeting^8^. Indeed, CHIKV and ZIKV have structural differences that lead to varied interactions with host cells and, consequently, different assemblies of the immune response^9^.

Previous research has shown that arbovirus cocirculation, particularly dengue virus (DENV), CHIKV, and ZIKV, is common in highly endemic regions^10,11^. Previous reports on arbovirus coinfection suggest that the disease commonly presents with mild clinical symptoms^12^, although severe manifestations, including Guillain-Barré syndrome, encephalitis, and meningoencephalitis, have been observed in rare cases. Severe manifestations have been associated with the presence of CHIKV and ZIKV in cerebrospinal fluid or brain tissue in fatal cases^13,14^, raising concerns that coinfection could exacerbate neurological outcomes, and to the hypothesis that coinfection could be a risk factor for severe and fatal outcomes in immunosuppressed adults^15^. Despite the detection of CHIKV and ZIKV in the cerebrospinal fluid, there is no clear evidence that coinfection enhances neuroinvasion, causing brain damage and neurological disorders. To address this gap, the current study evaluates the clinical characteristics of monoinfected and coinfected patients and is the first to assess immunological markers in patients with CHIKV and ZIKV.

## Results

### Patients with CHIKV and ZIKV coinfection exhibit no distinct clinical characteristics

Coinfection of ZIKV and CHIKV is a significant issue in Brazil, with a prevalence of 1.0%^3^. During study recruitment, out of more than 300 patients evaluated at the university hospital clinic for symptoms of arboviral disease, 12 patients were identified with concurrent CHIKV and ZIKV infections. This coinfected group had a median age of 39 years, and 75% were women, as shown in Table 1. These coinfected patients were compared to patients with only chikungunya (CHIKV-monoinfected group) or Zika (ZIKV-monoinfected group) infections. The monoinfected patients were selected from those seen at the same hospital and were matched for sex and age, with median ages of 34 for CHIKV and 43 years for ZIKV. Coinfected patients showed a median of 4 days of symptoms at recruitment, compared to medians of 2 days for CHIKV-monoinfected and 3 days for ZIKV-monoinfected patients.

**Table 1:** Clinical and epidemiological information from patients included in the study

Variables	Coinfected	CHIKV	ZIKV	*p*
	*n* = 12	*n* = 13	*n* = 13	
Age^a^				
	39 [28–60]	34 [21–46]	43 [37–57]	0.093
Sex (%)^b^				
Female	9 (75)	9 (71.2)	11 (78.6)	0.6479 ^c^
Cycle threshold in RT-qPCR				
CHIKV+ Ct	28.4 [20.2–37.4]	20.4 [18.5–21.7]	-	**<0.0001**^d^
ZIKV+ Ct	37.5 [36.7–37.7]	-	37.2 [36.7–37.7]	
Symptoms				
Fever	11 (91.7)	13 (100)	8* (61.5)	**0.018**
Arthralgia	11 (91.7)	13 (100)	12 (92.3)	0.576
Exanthema	9 (75)	4 (30.8)	7 (53.8)	0.086
Conjunctivitis	2 (16.7)	6 (46.1)	2 (15.4)	0.134
Myalgia	8 (66.7)	10 (76.9)	12 (92.3)	0.284
Retro-orbital pain	4 (33.3)	9 (69.2)	6 (46.1)	0.188
Lymphadenopathy	5 (41.7)	2 (15.4)	1 (7.7)	0.094
Medication used				
H1 receptor antagonists	1 (8.3)	0	4 (30.8)	0.057
NSAIDs	8 (66.7)	4 (30.8)	9 (69.2)	0.090
Analgesic	10 (83.3)	8 (61.5)	10 (76.9)	0.441
Antipyretic	7 (58.3)	6 (46.1)	8 (61.5)	0.708
Chronic co-morbidities				
Rhinitis	1 (8.3)	0	3 (23.1)	0.152
Asthma	0	0	2 (15.4)	0.131
Arterial hypertension	1 (8.3)	1 (7.7)	0	0.576
Diabetes	0	1 (7.7)	0	0.372

^a^data shown as median plus interquartile range (25%-75%).

^b^data shown as *n* plus percentage for group.

^c^Data tested with: Fisher’s exact test (for association comparisons); Mann-Whitney test (for continuous variables).

^d^Kruskal-wallis test followed by Dunn test (significant difference between CHIKV x ZIKV (*p* = 0.006)).

*The Chi-square posttest show the difference in ZIKV compared to CHIKV + ZIKV and CHIKV

Bold indicates statistical significative differences (*p* < 0.05).

The quantification of viral load by qPCR performed for molecular diagnostics showed that patients infected with CHIKV had lower Ct values, indicating higher viral load when compared to those infected with ZIKV, regardless of their infection status, indicating that ZIKV produces lower viral load than CHIKV (*p* < 0.0001, Kruskal-Wallis test). Moreover, CHIKV monoinfected patients produced significantly higher viral loads (lower Ct median) than those coinfected (*p* = 0.015, Mann-Whitney test).

Coinfected patients showed no differences in symptom presentation compared to those in the CHIKV- and ZIKV-monoinfected groups. Patients infected with ZIKV alone experienced a lower incidence of fever during the acute phase. Additionally, there were no differences in the presence of comorbidities or in the medications used during the disease among the groups. None of the coinfected patients reported persistent symptoms after the acute phase, similar to the monoinfected patients

### Coinfected patients exhibit a distinct inflammatory profile compared to monoinfected patients

**Fig. 1:**
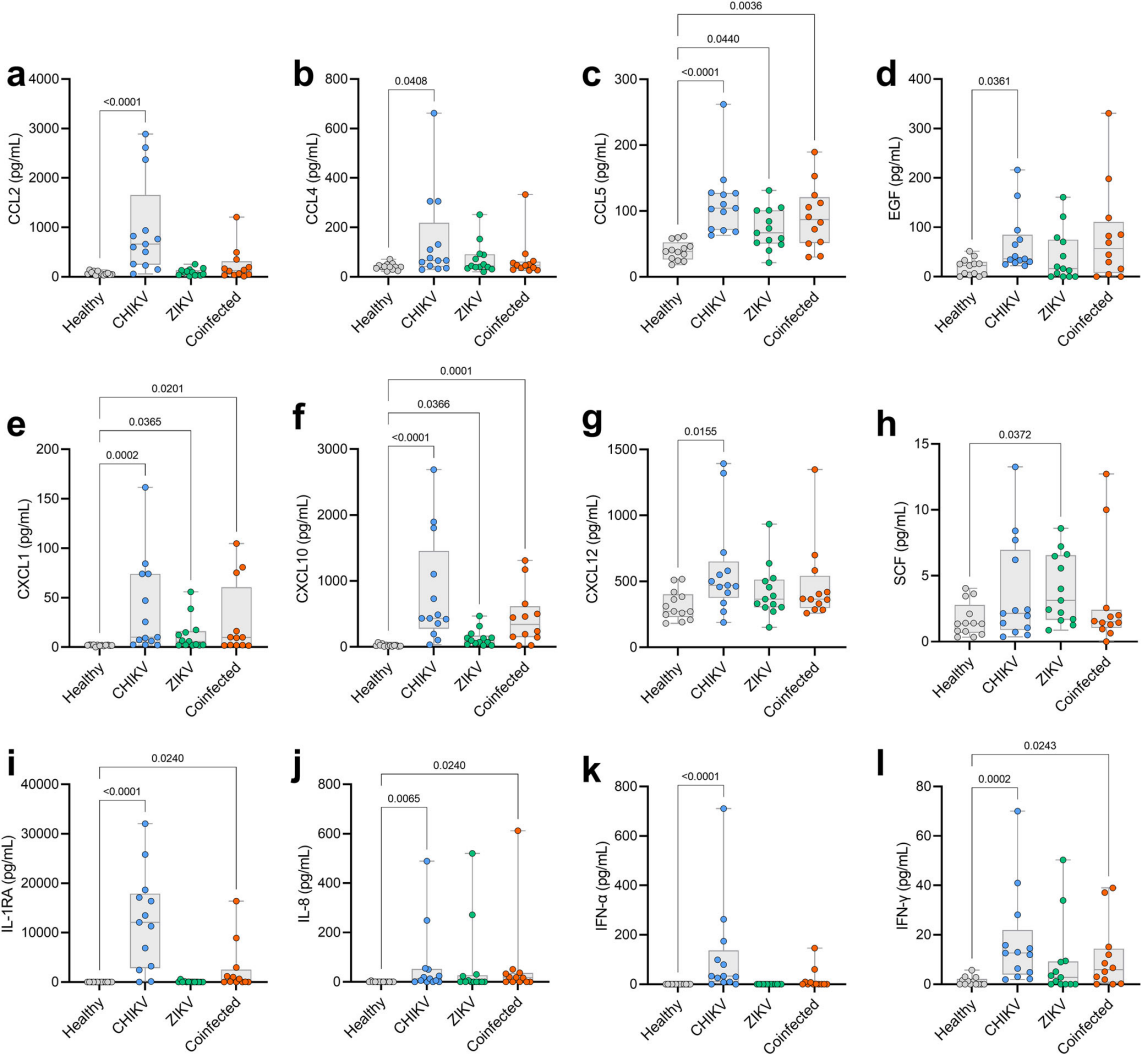
Differentially expressed cytokines, chemokines, and growth factors among patient groups: ZIKV (green) and CHIKV (blue) monoinfected, Coinfected (orange), and noninfected controls (grey). Of the 45 immune mediators quantified in patients’ sera, 12 were differentially expressed and are presented as follows: CCL2 (**a**), CCL4 (**b**), CCL5 (**c**), EGF (**d**), CXCL1 (**e**), CXCL10 (**f**), CXCL12 (**g**), SCF (**h**), IL-1RA (**i**), IL-8 (**j**), IFN-α (**k**), and IFN-γ (**l**). Each dot represents one participant; the boxes indicate the median and interquartile ranges (25%–75%), and the lines denote the minimum and maximum values. Comparisons were made using the Kruskal-Wallis test followed by Dunn’s test. The p-value is presented for each specific comparison.

We analyzed 45 cytokines, chemokines, and growth factors in the sera of patients and 13 matched noninfected controls. The analysis revealed that 12 of the 45 (26.7%) immunological mediators were differentially expressed in at least one patient group – CHIKV monoinfected, ZIKV monoinfected, or Coinfected – compared to noninfected controls. Specifically, these mediators include CCL2 (Fig. 1a), CCL4 (Fig. 1b), CCL5 (Fig. 1c), EGF (Fig. 1d), CXCL1 (Fig. 1e), CXCL10 (Fig. 1f), CXCL12 (Fig. 1g), SCF (Fig. 1h), IL-1RA (Fig. 1i), IL-8 (Fig. 1j), IFN-α (Fig. 1k), and IFN-γ (Fig. 1l).

Among these mediators, CCL2, CCL4, EGF, CXCL12, and IFN-α showed significantly elevated levels exclusively in CHIKV mono-infected patients compared to non-infected controls. Three mediators, IL-1RA, IL-8, and IFN-γ, exhibited significantly higher expression levels in both CHIKV-infected groups (monoinfected and coinfected), while three chemokines, CCL5, CXCL1, and CXCL10, demonstrated significantly elevated levels across all three infected groups (CHIKV monoinfected, ZIKV monoinfected, and coinfected). One growth factor, SCF, showed significantly elevated levels only in the ZIKV monoinfected group. No correlations were found between Ct values and immune mediators.

**Fig. 2:**
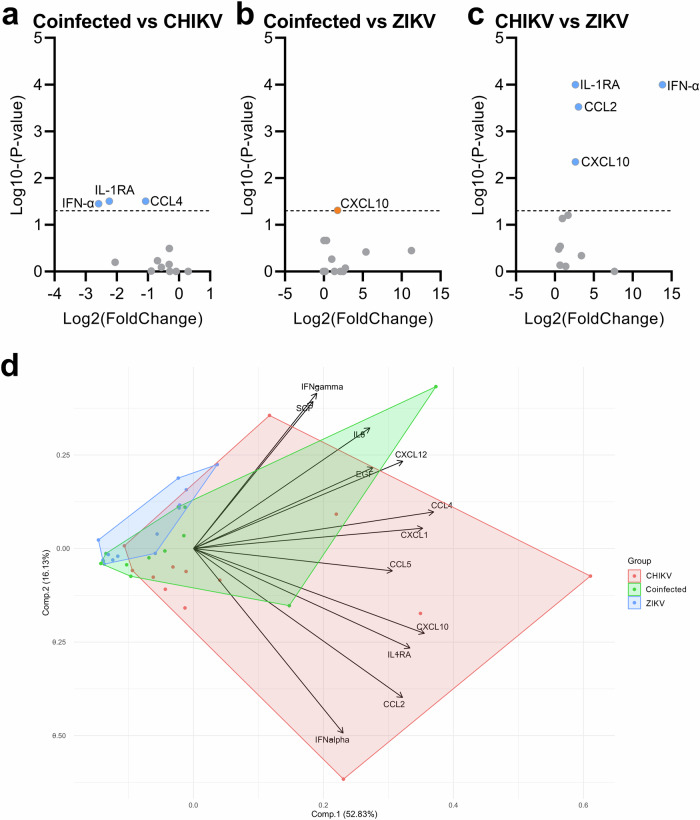
Exploration of specific differences among groups of infected patients. Comparisons were initially made between levels of immune mediators in pairs of groups, revealing differences between Coinfected versus CHIKV-monoinfected (**a**), Coinfected versus ZIKV mono-infected (**b**), and CHIKV versus ZIKV monoinfected groups (**c**). The volcano plots display differences based on the fold change of quantified cytokines and p-values from multiple comparisons tests (shown as Log_2_ and Log_10_, respectively). **d** Principal component analysis of the 12 differentially expressed cytokines across the three infected groups. The PCA biplot, based on the two principal components (accounting for approximately 70% of the variance), illustrates the overlap of the three patient clusters and the relationships among cytokines, as indicated by the loadings on the graph.

To further explore these 12 differentially expressed mediators, we performed two analyses. First, the comparative analyses indicated that the CHIKV monoinfected group had significantly higher levels of IL-1RA, IFN-α, and CCL4 compared to the coinfected group (Fig. 2a), while the coinfected group displayed significantly higher expression of CXCL10 compared to the ZIKV monoinfected group (Fig. 2b). The CHIKV monoinfected group also showed higher levels of CCL2, CXCL10, and IFN-α than the ZIKV monoinfected group (Fig. Fig. 2c). Additionally, the principal component analysis revealed overlapping groups with no clear segregation (Fig. 2d), displaying greater dispersion in the direction of immune mediators for the CHIKV monoinfected group. In contrast, the coinfected and ZIKV monoinfected groups showed moderate to low dispersion, respectively. Additional correlation analyses between Ct values and cytokines, chemokines, and growth factors did not reveal any significant positive or negative correlations. Similarly, no significant correlations between age and immune mediators were observed.

**Fig. 3:**
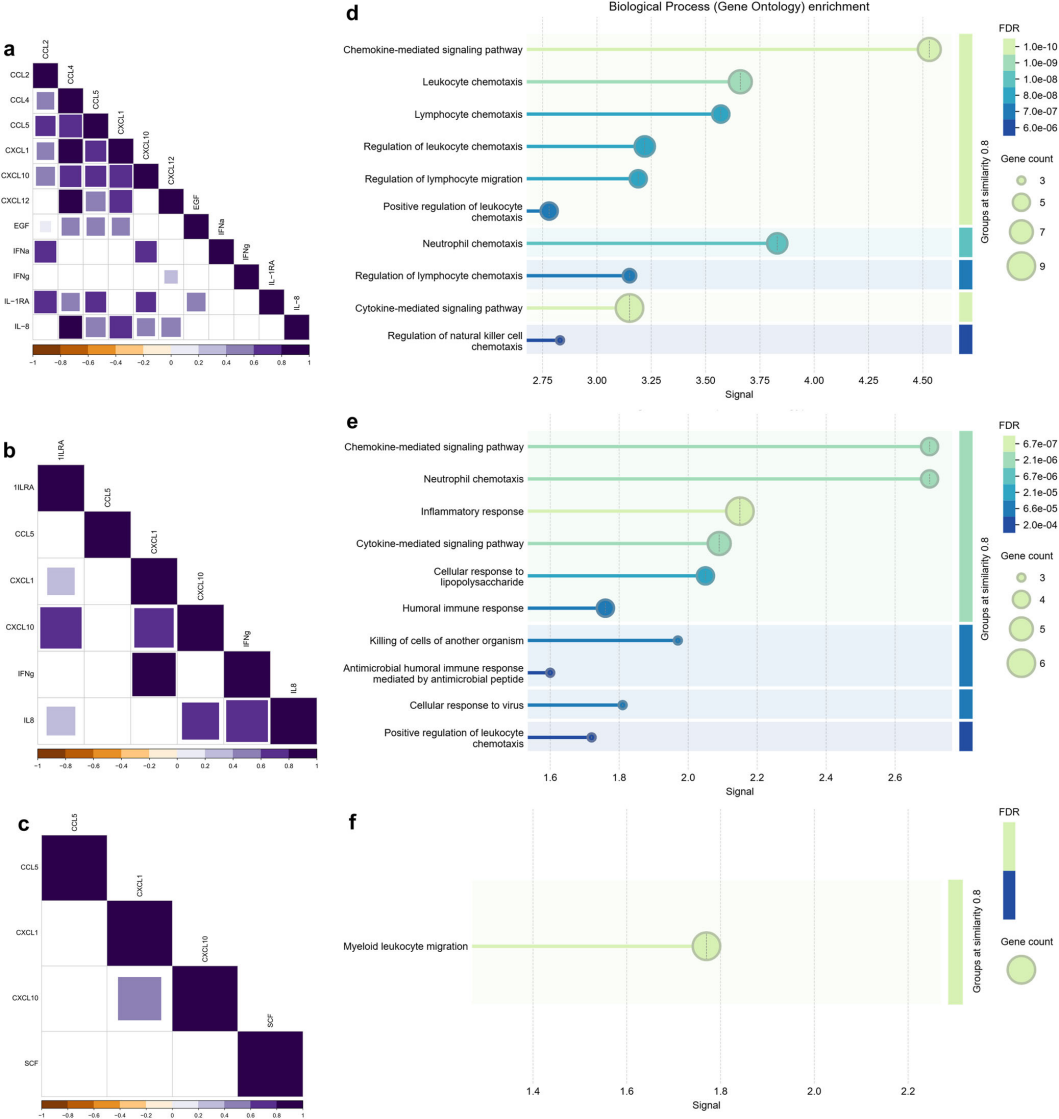
Correlation and enrichment analysis of serological mediators in infected groups. The correlations among differentially expressed cytokines, chemokines, and growth factors in the CHIKV (**a**), Coinfected (**b**), and ZIKV (**c**) groups exhibit distinct patterns, including only positive and significant correlations (with purple intensity indicating the strength of positive values). Non-significant correlations are not shown. Data were analyzed using Spearman’s correlation. An enrichment analysis of protein-protein interaction networks was performed using STRING. Biological processes are depicted for each group: CHIKV (**d**), Coinfected (**e**), and ZIKV (**f**). The false discovery rate (FDR) was calculated using the Benjamini–Hochberg method, and graded p-values are displayed using a green-blue gradient. The signal measure represents the weighted harmonic mean between the observed/expected ratio and -log(FDR). The number of proteins included in the analysis is indicated by circle size.

In a complementary manner, correlation analysis was performed for all differentially expressed cytokines, chemokines, and growth factors for each group. The results show that in the CHIKV monoinfected group, among all 12 altered mediators, the chemokines exhibit strong correlations for themselves, with clustering observed for CCL4, CCL5, CXCL1, CXCL10, and IL-8 (Fig. 3a). Conversely, the coinfected group exhibits similar results to that of the CHIKV monoinfected group, but to a lesser extent, mainly involving the mediators CXCL10, IL-1RA, and IL-8 (Fig. 3b). Among the four immune mediators in the ZIKV monoinfected group, there is only a significant moderate correlation between the chemokines CXCL1 and CXCL10 (Fig. 3c). The enrichment analysis of biological processes using STRING protein-protein association networks shows that the immune mediators in the CHIKV monoinfected group are mainly related to chemokine-mediated signaling pathways and neutrophil, leukocyte, and lymphocyte chemotaxis (Fig. 3d). Moreover, the interactions of immune mediators in coinfected patients also show, besides more modest, enrichment of chemokine-mediated signaling pathways and neutrophil chemotaxis, as well as inflammatory and cytokine signaling pathways (Fig. 3e). Finally, the enrichment analysis of the ZIKV monoinfected group shows the presence of the myeloid leukocyte migration pathway (Fig. 3f).

## Discussion

In this study, we evaluated a case series of patients coinfected with CHIKV and ZIKV, comparing them to well-matched groups of patients’ monoinfected with each virus and noninfected subjects. In summary, no distinct clinical characteristics were observed in coinfected patients compared to those with only CHIKV or ZIKV infections. Viral load analysis revealed lower ZIKV loads in coinfected patients, regardless of mono or coinfection. Additionally, immune profiling identified an inflammatory signature in coinfected individuals that was intermediate compared to monoinfections, differing from monoinfected patients in specific cytokine expressions.

Our results show that CHIKV and ZIKV coinfected patients exhibited no significant differences in clinical manifestations compared to those with monoinfections, except for a lower frequency of fever in ZIKV-monoinfected patients, a well-documented characteristic of ZIKV infection^16^. This may be related to the lower viral loads found for ZIKV in both monoinfected and coinfected patients, leading to a less intense immune response in the infected host. Moreover, none of the patients in any group developed severe or persistent symptoms after the acute phase. ZIKV infection is well-known for not producing chronic disease, but previous reports suggest it could be associated with long-term neurological manifestations^17^. On the other hand, CHIKV infection has a prevalence of chronic symptoms in 40% of patients^18^, which was not observed in the present study. Based on our results, we hypothesize that the frequency of complications in CHIKV and ZIKV coinfections is comparable to that observed in CHIKV and ZIKV mono-infections. These complications may be due to individual patient factors that warrant further investigation and are beyond the scope of this study.

We rigorously screened over 300 individuals, ensuring strict patient selection to create well-matched groups to provide confidence in the robustness of our findings. Although, the rate of ZIKV and CHIKV coinfection in our cohort was 4%, three-times higher than the reported 1%^3^, studies of this nature are challenging and require the screening of many patients and strict selection criteria to produce meaningful data. Indeed, we recruited patients during an outbreak in a geographical region that is part of a cluster experiencing intense co-transmission of CHIKV and ZIKV^19^. This co-occurrence could lead to more intense transmission of both viruses, a phenomenon not clearly observed in other studies.

To our knowledge this is the first study to provide an extensive cytokine profile for CHIKV and ZIKV coinfected patients, showing that coinfection leads to an intermediate inflammatory profile between CHIKV and ZIKV monoinfections. A previous report evaluating eight cytokines and chemokines among patients with CHIKV and ZIKV coinfection and monoinfections found similarities among all groups, except that ZIKV-monoinfected patients exhibited more intense clinical manifestations^20^. Conversely, we observed that CHIKV infection triggered a robust immune response, upregulating eleven immune markers, while ZIKV infection resulted in a modest increase in only three immune mediators to lower symptomatology. This aligns with previously published data suggesting that CHIKV and ZIKV infections elicit robust and less robust immune responses, respectively^21,22^. However, it is necessary to emphasize that differences could be due to host genetic characteristics that lead to variations in the immune response. In this regard, our group has demonstrated that certain genetic characteristics in humans from the study population, when infected with arboviruses or other infectious and parasitic diseases, could lead to different disease progression and evolution^5,23–25^.

Indeed, it is well known that CHIKV proteins can interact with more host factors than ZIKV, which could contribute to a more diverse response to infection^9^, as observed in the present study. Therefore, it is possible that concomitant ZIKV and CHIKV infection leads to viral interference rather than synergy^26^, resulting in a more modest immune response, as previously observed by reduced viral loads in coinfections among CHIKV, DENV, and ZIKV^27^. Corroborating this, our results show that coinfected patients had a higher average Ct value for CHIKV detection than patients infected with CHIKV alone, which may indicate interference by ZIKV leading to a lower CHIKV viral load in concomitant infections. Furthermore, this viral interference and lower immune response could be related to similar clinical manifestations, with no severe cases and no worse prognosis observed in coinfected patients, as indicated by our results. However, more studies are necessary to clarify this, especially infection models that could determine whether there is interference between CHIKV and ZIKV.

As expected, CXCL10 levels were elevated in all groups, as this chemokine is upregulated by type I and II IFNs following viral infections and plays a key role in the pathogenesis of viral infections. This study corroborates previous finding of CXCL10 elevation during the acute phase of CHIKV and ZIKV infections^28^. Specific comparisons show that the coinfected and CHIKV groups displayed higher levels of CXCL10 than the ZIKV group, indicating a more robust inflammatory profile in these groups. However, despite previous associations of this cytokine with severe disease in arboviral infections^29^, severe cases were not observed in the present study.

Moreover, the CHIKV monoinfected group exhibited higher levels of IL-1RA, IFN-α, CCL2, and CCL4 than the other groups. These markers are linked to the acute antiviral response and are related to different prognoses of CHIKV, ZIKV, and other arbovirus infections^30–32^. However, additional analyses did not reveal a specific segregation of the immune response among the groups. ZIKV-infected patients exhibited a less intense immune response, primarily involving three chemokines present across all groups – CXCL10, CCL5, and CXCL1 – which have been previously associated with ZIKV infection^33^. These patients also exhibited higher levels of SCF, a growth factor released in inflammatory conditions and associated with the protection of the fetus in ZIKV-infected pregnant women^34^. Indeed, the enrichment analysis of protein–protein interactions revealed a more pronounced response of chemokine-mediated chemotaxis activity in patients with CHIKV monoinfection or coinfection, including neutrophil attraction, which could act as protection or lead to disease exacerbation^35^. Additionally, the pronounced presence of other pathways linked to cytokine and inflammatory responses in coinfected patients corroborates the elicited differential response in coinfection with CHIKV and ZIKV.

In conclusion, CHIKV and ZIKV coinfection resulted in mild clinical symptoms, similar to those seen in monoinfections, and presented a profile of circulating immune mediators more robust than ZIKV infection but more modest than CHIKV infection. Our study makes an important contribution to the limited research on coinfection and its impact on disease severity and host responses. We recognize the limitation in sample size of this study to draw strong conclusion, however, it provides valuable information regarding profiles of immune mediators produced during acute phase of CHIKV, ZIKV and coinfection with these viruses. The reported data adds to the field and to further studies on the pathogenesis of arboviruses infections and coinfections.

## Materials And Methods

### Ethics statement

The research was developed according to national guidelines and was approved by the Ethics Committee of the Federal University of Sergipe (protocol number 1.486.302). The participants provided written informed consent after receiving verbal information about the study.

### Study design, sampling, and diagnostics

**Fig. 4:**
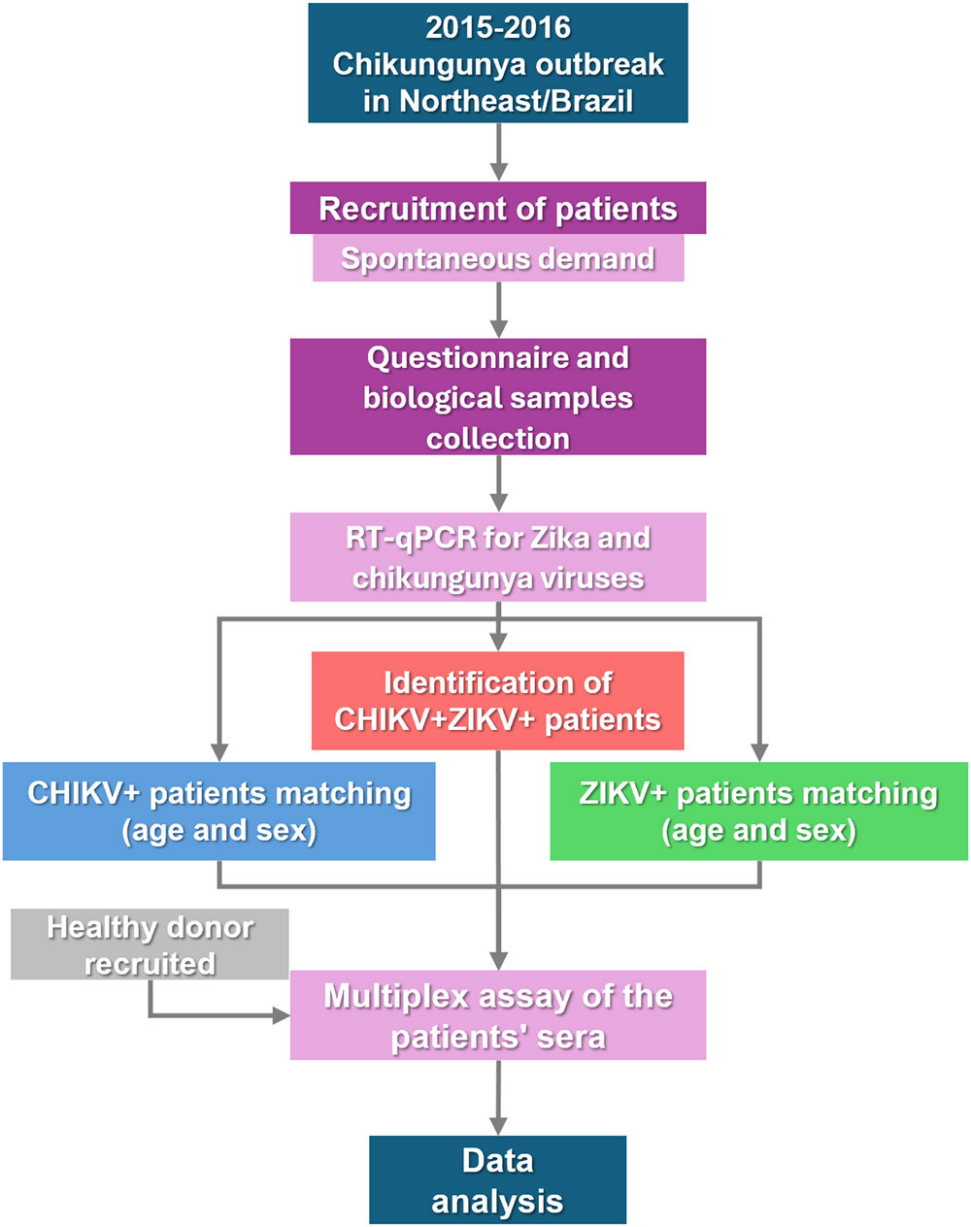
Study Rationale. Timeline summarizing the study rationale and the steps of patient recruitment, blood collection, sample processing, data acquisition, and analysis.

This research is a cross-sectional observational study involving patients with concomitant infections of chikungunya and Zika viruses. The study was approved by the local ethics committee and informed consent was obtained. Recruitment occurred throughout 2016 at the University Hospital of the Federal University of Sergipe. Participants were divided into one noninfected control group and three primary patient groups: the CHIKV monoinfected group, the ZIKV monoinfected group, and the Coinfected group (Fig. 4).

All patients were adults who spontaneously reported to the hospital with signs and symptoms of arboviral infection. The selection criteria for inclusion in this study were based on positive qPCR results for CHIKV and/or ZIKV and patients who were symptomatic for less than 14 days. A total of 300 patients were screened by qRT-PCR using previously reported protocols and 38 participants were selected and divided into three groups: the CHIKV monoinfected group (*n* = 13), the ZIKV monoinfected group (*n* = 13), and the CHIKV and ZIKV coinfected group (*n* = 12). The control group (*n* = 13) consisted of well-matched noninfected individuals recruited based on the lack of clinical symptoms and a negative history of arboviral infection, who tested negative for IgG and IgM for both CHIKV and ZIKV.

Data were collected for all groups at the initial assessment included age, sex, duration and type of symptoms, including the presence of conjunctivitis, exanthema, fever, myalgia, lymphadenopathy, and retro-orbital pain. The frequency of treatment with analgesics, NSAID, antihistaminic, and antipyretic and diagnosis of comorbidities were also documented. Blood samples were collected from all participants during their initial visit and stored frozen until the assay execution.

The exclusion criteria were patients with cancer, or in chronic use of immune modulators, or those diagnosed with other inflammatory or infectious diseases. All patients included in this study achieved clinical cure without developing complications from the arboviral infection or requiring hospitalization.

### Detection of chikungunya and Zika virus

Viral RNA was isolated from serum or plasma samples utilizing the QIAamp Viral RNA Mini Kit (QIAGEN, Germany) in accordance with the manufacturer’s protocols. Subsequently, reverse transcription followed by quantitative real-time polymerase chain reaction (qRT-PCR) was performed using the SuperScript® III Platinum® One-Step qRT-PCR System from Invitrogen (Thermo Fisher Scientific, USA). Specific primers and probes targeting chikungunya^36^ and Zika^37^ viruses were sourced from Integrated DNA Technologies (IDT, USA). Amplification was executed on an ABI 7500 FAST system (Applied Biosystems, USA). Samples yielding a cycle threshold (CT) value of 38 or below were classified as positive for either Chikungunya or Zika virus infection^36^.

### Quantification of immunological mediators

The immune response markers were quantified using the ProcartaPlex™ Human Cytokine/Chemokine/Growth Factor Panel 1, 45plex kit (Thermo Fisher, USA). The analysis was conducted according to the manufacturer’s instructions using a Luminex® 100 system (Merck Millipore, USA). Results are expressed in picograms per milliliter (pg/mL). The following were the analytes studied: Th1/Th2: granulocyte-macrophage colony-stimulating factor (GM-CSF), interferon γ (IFN-γ), interleukin 1β (IL-1β), IL-2, IL-4, IL-5, IL-6, IL-8, IL-12p70, IL-13, IL-18, tumor necrosis factor α (TNF-α); Th9/Th17/Th22/Treg: IL-9, IL-10, IL-17A, IL-21, IL-22, IL-23, IL-27; Inflammatory cytokines: IFN-α, IL-1α, IL-1 receptor antagonist (IL-1RA), IL-7, IL-15, IL-31, leukemia inhibitory factor (LIF), Lymphotoxin α (LT-α or TNF-β); Chemokines: C-C motif chemokine ligand 11 (CCL11 or Eotaxin), C-X-C motif chemokine ligand 1 (CXCL1), CXCL10 (or IP-10), CCL2 (or MCP-1), CCL3 (or MIP-1α), CCL4 (or MIP-1β), CCL5 (or RANTES), CXCL12 (or SDF-1α); Growth factors: brain-derived neurotrophic factor (BDNF), epidermal growth factor (EGF), fibroblast growth factor 2 (FGF-2), hepatocyte growth factor (HGF), nerve growth factor β (β-NGF), platelet derived growth factor subunit B (PDGF-BB), placental growth factor 1 (PIGF-1), stem cell factor (SCF), vascular endothelial growth factor A (VEGF-A), VEGF-D.

### Data processing and statistical analysis

All statistical analyses were conducted using GraphPad Prism 9 and R version 4.3. Differences were considered statistically significant when *p* < 0.05 in a two-tailed analysis. The STROBE guidelines were followed to construct this report (checklist v4)^38^. For categorical variables, Pearson’s chi-squared test, followed by post-hoc analysis, was employed to identify differences among groups, using the ‘chisq.posthoc.test’ package in R.

For continuous numerical data, the normality of the data was first assessed using the Shapiro-Wilk test. Subsequently, the Kruskal-Wallis test, followed by Dunn’s test with adjustments for multiple comparisons, was used to compare groups. For selected immune mediators, volcano plots were generated by plotting the log_2_ fold change against the negative log_10_ p-value for each comparison. Additionally, principal component analysis (PCA) was performed and plotted using the ‘ggfortify’ package. To understand the relationships among immune mediators, correlations were calculated using Spearman’s rank correlation (rho) and plotted using the ‘corrplot’ package. Furthermore, analysis of protein–protein interactions and biological enrichment was performed using STRING^39^.

## Supplementary information

STROBE-checklist-v4-cross-sectional

## Data Availability

All data is presented in the paper. Additional data could be obtained with the corresponding author upon requisition.

## References

[1] Huang, Y. J. S., Higgs, S. & Vanlandingham, D. L. Emergence and re-emergence of mosquito-borne arboviruses. *Curr. Opin. Virol.* **34**, 104–109 (2019). 30743191 10.1016/j.coviro.2019.01.001

[2] Mayer, S. V., Tesh, R. B. & Vasilakis, N. The emergence of arthropod-borne viral diseases: A global prospective on dengue, chikungunya and zika fevers. *Acta Trop.* **166**, 115–163 (2017). 10.1016/j.actatropica.2016.11.020 PMC5203945 27876643

[3] Ahmed, S. et al.Global Prevalence of Zika and Chikungunya Coinfection: A Systematic Review and Meta-Analysis. *Dis.* **12**, 31 (2024). 10.3390/diseases12020031 PMC10888207 38391778

[4] de Almeida, P. N. G. et al. Detection of Chikungunya Virus RNA in Cerebrospinal Fluid of Patients with Severe Neurological Disorders. *SN Compr. Clin. Med.* **5**, 50 (2023).

[5] Santos, C. N. O. et al. Association between genetic variants in TREM1, CXCL10, IL4, CXCL8 and TLR7 genes with the occurrence of congenital Zika syndrome and severe microcephaly. *Sci. Rep.* **13**, 3466 (2023). 36859461 10.1038/s41598-023-30342-3 PMC9975867

[6] Silva, M. M. O. et al. Risk of chronic arthralgia and impact of pain on daily activities in a cohort of patients with chikungunya virus infection from Brazil. *Int. J. Infect. Dis.* **105**, 608–616 (2021). 33684559 10.1016/j.ijid.2021.03.003

[7] Lee, L. J., Komarasamy, T. V., Adnan, N. A. A., James, W. & Balasubramaniam RMT, V. Hide and Seek: The Interplay Between Zika Virus and the Host Immune Response. *Fronti. Immunol*. **12**, 750365 (2021). 10.3389/fimmu.2021.750365 PMC8566937 34745123

[8] Ravindran, S. & Lahon, A. Tropism and immune response of chikungunya and zika viruses: An overview. *Cytokine* **170**, 156327 (2023). 37579710 10.1016/j.cyto.2023.156327

[9] Wichit, S. et al.Chikungunya and zika viruses: Co-circulation and the interplay between viral proteins and host factors. *Pathogens* **10**, 448 (2021). 33918691 10.3390/pathogens10040448 PMC8068860

[10] Lobkowicz, L. et al. The frequency and clinical presentation of Zika virus coinfections: A systematic review. *BMJ J Glob. Health* **5**, e002350 (2020). 10.1136/bmjgh-2020-002350 PMC7228501 32381652

[11] Santos, L. L. M., de Aquino, E. C., Fernandes, S. M., Ternes, Y. M. F. & de Feres, V. C. R. Dengue, chikungunya, and Zika virus infections in Latin America and the Caribbean: a systematic review. *Revista Panamericana de Salud Publica/Pan American Journal of Public Health* **47**, e34 (2023). 36788963 10.26633/RPSP.2023.34 PMC9910557

[12] Zambrano, H. et al. Case report: Zika virus and chikungunya virus coinfections: A series of three cases from a single center in Ecuador. *Am. J. Tropical Med. Hyg.* **95**, 894–896 (2016). 10.4269/ajtmh.16-0323 PMC5062796 27402518

[13] Brito, C. A. A., Azevedo, F., Cordeiro, M. T., Marques, E. T. A. & Franca, R. F. O. Central and peripheral nervous system involvement caused by Zika and chikungunya coinfection. *PLoS Negl. Trop. Dis.* **11**, e0005583 (2017). 28704365 10.1371/journal.pntd.0005583 PMC5509110

[14] Acevedo, N. et al. Zika virus, chikungunya virus, and dengue virus in cerebrospinal fluid from adults with neurological manifestations, Guayaquil, Ecuador. *Front Microbiol.* **8**, 42 (2017). 28174559 10.3389/fmicb.2017.00042 PMC5258761

[15] Darrigo, L. G., de Sant'Anna Carvalho, A. M. & Machado, C. M. Chikungunya, Dengue, and Zika in Immunocompromised Hosts. *Curr. Infect. Dis. Rep.* **20**, 5 (2018). 29551005 10.1007/s11908-018-0612-2 PMC5857271

[16] Masmejan, S. et al. Zika Virus. *Pathogens* **9**, 1–14 (2020).

[17] de Azevedo, M. B. et al. Neurologic manifestations in emerging arboviral diseases in Rio de Janeiro city, Brazil, 2015-2016. *Rev. Soc. Bras. Med Trop.* **51**, 347–351 (2018). 29972566 10.1590/0037-8682-0327-2017

[18] Ng, W. H., Amaral, K., Javelle, E. & Mahalingam, S. Chronic chikungunya disease (CCD): clinical insights, immunopathogenesis and therapeutic perspectives. *QJM: Int. J. Med.* **117**, 489–494 (2024). 10.1093/qjmed/hcae028 PMC11290245 38377410

[19] Gardini Sanches Palasio, R., Marques Moralejo Bermudi, P., Luiz de Lima Macedo, F., Reis Santana, L. M. & Chiaravalloti-Neto, F. Zika, chikungunya and co-occurrence in Brazil: space-time clusters and associated environmental–socioeconomic factors. *Sci. Rep.* **13**, 18026 (2023). 37865641 10.1038/s41598-023-42930-4 PMC10590386

[20] Sánchez-Arcila, J. C. et al. Clinical, Virological, and Immunological Profiles of DENV, ZIKV, and/or CHIKV-Infected Brazilian Patients. *Intervirology* **63**, 33–45 (2020). 32966990 10.1159/000510223

[21] de Barros, J. B. S. et al. Acute Zika virus infection in an endemic area shows modest proinflammatory systemic immunoactivation and cytokine-symptom associations. *Front Immunol.* **9**, 821 (2018). 29774022 10.3389/fimmu.2018.00821 PMC5943559

[22] Venugopalan, A., Ghorpade, R. P. & Chopra, A. Cytokines in acute chikungunya. *PLoS One* **9**, e111305 (2014). 25343623 10.1371/journal.pone.0111305 PMC4208842

[23] Bezerra-Santos, M. et al. sTREM-1 and TNF-α levels are associated with the clinical outcome of leprosy patients. *Front Med (Lausanne)* **10**, 1177375 (2023). 37457576 10.3389/fmed.2023.1177375 PMC10339318

[24] Franco, K. G. S. et al. Association of IL-9, IL-10, and IL-17 Cytokines With Hepatic Fibrosis in Human Schistosoma mansoni Infection. *Front Immunol.* **12**, 1–11 (2021). 10.3389/fimmu.2021.779534 PMC8712476 34970264

[25] Santos, C. N. O. et al. Association Between Zika Virus Microcephaly in Newborns With the rs3775291 Variant in Toll-Like Receptor 3 and rs1799964 Variant at Tumor Necrosis Factor-α Gene. *J. Infect. Dis.* **220**, 1797–1801 (2019). 31352487 10.1093/infdis/jiz392

[26] Du, Y., Wang, C. & Zhang, Y. Viral Coinfections. *Viruses* **14**, 2645 (2022). 36560647 10.3390/v14122645 PMC9784482

[27] Mercado-Reyes, M. et al. Dengue, chikungunya and zika virus coinfection: Results of the national surveillance during the zika epidemic in colombia. *Epidemiol. Infect.* **147**, e77 (2019). 30869010 10.1017/S095026881800359X PMC6518562

[28] Naveca, F. G. et al. Analysis of the immunological biomarker profile during acute zika virus infection reveals the overexpression of CXCL10, a chemokine linked to neuronal damage. *Mem. Inst. Oswaldo Cruz* **113**, e170542 (2018). 29768624 10.1590/0074-02760170542 PMC5961926

[29] Lin, T. et al. Cxcl10 signaling contributes to the pathogenesis of arthritogenic alphaviruses. *Viruses* **12**, 1252 (2020). 33147869 10.3390/v12111252 PMC7692144

[30] de-Oliveira-Pinto, L. M. et al. Profile of circulating levels of IL-1Ra, CXCL10/IP-10, CCL4/MIP-1β and CCL2/MCP-1 in dengue fever and parvovirosis. *Mem. Inst. Oswaldo Cruz* **107**, 48–56 (2012). 22310535 10.1590/s0074-02762012000100007

[31] Locke, M. C. et al. Interferon Alpha, but Not Interferon Beta, Acts Early To Control Chronic Chikungunya Virus Pathogenesis. *J. Virol.* **96**, e0114321 (2022). 34668781 10.1128/JVI.01143-21 PMC8754211

[32] Ruiz Silva, M., Van Der Ende-Metselaar, H., Mulder, H. L., Smit, J. M. & Rodenhuis-Zybert, I. A. Mechanism and role of MCP-1 upregulation upon chikungunya virus infection in human peripheral blood mononuclear cells. *Sci. Rep.* **6**, 32288 (2016). 27558873 10.1038/srep32288 PMC4997611

[33] Espino, A. et al. The mechanisms underlying the immune control of Zika virus infection at the maternal-fetal interface. *Front Immunol.* **13**, 1000861 (2022). 36483552 10.3389/fimmu.2022.1000861 PMC9723234

[34] Kam, Y. W. et al. Specific biomarkers associated with neurological complications and congenital central nervous system abnormalities from Zika virus-infected patients in Brazil. *J. Infect. Dis.* **216**, 172–181 (2017). 28838147 10.1093/infdis/jix261 PMC5853428

[35] Muralidharan, A., Patrick Reid, S. & Groseth, A. cells Complex Roles of Neutrophils during Arboviral Infections. 10.3390/cells (2021). 10.3390/cells10061324 PMC8227388 34073501

[36] Lanciotti, R. S. et al. Chikungunya Virus in US Travelers Returning from India, 2006. *Emerg. Infect. Dis.* **13**, 764–767 (2007). 17553261 10.3201/eid1305.070015 PMC2738459

[37] Faye, O. et al. Quantitative real-time PCR detection of Zika virus and evaluation with field-caught Mosquitoes. *Virol. J.* **10**, 311 (2013). 24148652 10.1186/1743-422X-10-311 PMC4016539

[38] Von Elm, E. et al. *The Strengthening the Reporting of Observational Studies in Epidemiology (STROBE) Statement: Guidelines for Reporting Observational Studies*. www.thelancet.com 370 www.plosmedicine.org (2007). 10.1136/bmj.39335.541782.AD PMC2034723 17947786

[39] Szklarczyk, D. et al. The STRING database in 2023: protein-protein association networks and functional enrichment analyses for any sequenced genome of interest. *Nucleic Acids Res.* **51**, D638–D646 (2023). 36370105 10.1093/nar/gkac1000 PMC9825434

